# The Preparation of N, P-Doped NiSe Nanorod Electrode Materials on Nickel Foam Using the Microwave Method for High-Performance Supercapacitors

**DOI:** 10.3390/molecules29133224

**Published:** 2024-07-07

**Authors:** Zhen Lu, Hongjie Kang, Qianwen Duan, Chao Lv, Rui Liu, Feng Feng, Haidong Zhao

**Affiliations:** 1 School of Chemistry and Chemical Engineering, Shanxi Datong University, Datong 037009, China; luzhen2024@sxdtdx.edu.cn (Z.L.); 15603450662@163.com (H.K.); 18834839312@163.com (Q.D.); liurui198689@163.com (R.L.); 2 School of Coal Engineering, Shanxi Datong University, Datong 037009, China; lvchao0711@126.com

**Keywords:** N, P doped, microwave method, supercapacitors

## Abstract

Transition metal selenides have the leading position in the field of energy storage and conversion due to their high theoretical capacity, good electrical conductivity, and cycling stability. Nickel is widely used for the construction of positive electrodes in devices due to its good conductivity, variable valence state, and ideal redox activity. NiSe materials have high internal resistance and are prone to volume change during charging and discharging, thus affecting the practical application of this electrode material, and the reported NiSe materials have not achieved a more desirable capacity value. Therefore, in this study, N, P-NiSe nanoelectrode materials were prepared using nickel foam as the nickel source and hexachlorocyclotriphonitrile as the nitrogen and phosphorus dopant using an efficient, energy-saving, and simple microwave method. It was also characterised by XRD and XPS to confirm the successful preparation of N, P-NiSe materials. In addition, the material yielded a high capacitance value (3184 F g^−1^) and good cycling stability (72% of the initial capacitance value was retained after 4000 cycles) in electrochemical tests. To demonstrate its excellent suitability for practical applications, an asymmetric supercapacitor was assembled using N, P-NiSe as the anode and activated carbon as the cathode. At an operating voltage of 1.6 V, the device achieved an energy density of 289.06 Wh kg^−1^ and a power density of 799.26 W kg^−1^ and retained 80% of its initial capacity after 20,000 cycles.

## 1. Introduction

Energy storage technology represents one of the most effective solutions to the growing energy crisis and environmental protection problems facing the world today [1]. Among the commonly used green energy storage technologies, supercapacitors have been widely noticed and researched due to their excellent capacitance storage capacity and cycle stability [2,3]. The key technology for the development of supercapacitors is the development of high-performance electrode materials [4,5,6,7]. Currently, the most commonly used electrode materials include carbon materials [8,9,10], polyaniline (PANI) [11], polypyrrole (PPy) [12], transition metal oxides [13], manganese dioxide and other active electrode materials [14]. However, the electrochemical properties of these electrode materials are not ideal. Therefore, the design and development of high-performance supercapacitor electrode materials is still a crucial topic in the field of green energy storage.

Nickel selenide, a semiconductor material, has received considerable attention in the fields of catalysis and energy storage due to its significant electronic and magnetic properties [15,16,17]. In 2012, Gao et al. prepared NiSe using a hydrothermal method and used it as a catalyst for electrochemical hydrogen production [18]. In 2017, Tian et al. prepared NiSe nanorods using a hydrothermal method, obtaining a specific capacitance of 6.81 F cm^−2^ (current density 5 mA cm^−2^) and demonstrating their good cycling stability. Furthermore, the capacitance retention was 90.09% after 3000 cycles (current density 3.6 A g^−1^) [19]. Younas et al. successfully prepared Ni_0.85_Se nanosheets. The hexagonal-phase structure of Ni_0.85_Se nanosheets exhibited excellent electrochemical performance in supercapacitors, as demonstrated with a fast microwave method. The nanosheet morphology shortened the diffusion of the ion transfer paths, thereby increasing the reaction kinetics. The assembled device exhibited a high energy density of 63.5 Wh kg^−1^ and excellent cycling performance (95% of the starting capacitance value after 8000 cycles) [20].

However, single nickel selenide materials often do not exhibit desirable capacitance values, and researchers have attempted to make greater progress by introducing heteroatoms to modify their performance [21,22,23]. Liu et al. prepared P-NiSe_2_ using a hydrothermal method. The results of electrochemical tests demonstrated that P doping could markedly enhance the HER-catalysed activity of NiSe_2_ nanomaterials and reduce their hydrogen precipitation reaction overpotentials [24]. Yan et al. demonstrated that N doping disrupted the octahedral coordination environment of the Ni atoms in NiSe, resulting in a decrease in the simplicity of orbitals and an upward band centre shift. This improved interfacial charge transfer kinetics and enhanced redox kinetics. The assembled Li-S batteries exhibited high performance (682.6 mA h g^−1^ at 5 C) and a low capacity decay rate, with the inclusion of N-NiSe [25]. In 2022, Wang prepared NiSe-RGo using a hydrothermal method. This method resulted in the homogeneous distribution of NiSe on graphene, which was beneficial for the electrochemical performance of the battery. This resulted in faster charge transport and the diffusion of NiSe-RGO nanohybrids, as well as an abundance of NiSe-RGO with a high capacitance value (781 C g^−1^ at 1 A g^−1^) and good cycling stability (90% of the initial capacitance after 5000 cycles) [26]. It can be concluded that doping with heteroatoms can alter the electrochemical properties of composites, demonstrating great potential for energy storage. The incorporation of heteroatoms not only alters the lattice structure to a greater extent, thereby exposing more active sites, but also induces stronger electronic interactions and optimises its free energy. Secondly, doping other elements can regulate the microstructure of the electrode material and prevent the structure from collapsing and affecting its performance during the electrolysis process.

In this study, nickel foam (NF) served as the nickel source, while hexachlorocyclotriphosphonitrile (HCCP) was employed as the nitrogen–phosphorus source. These components were utilised in the synthesis of nitrogen–phosphorus-doped nickel selenide (N, P-NiSe) electrode materials with self-supported structures. The self-supported structure reduced the loss of electron transport in the bonding agent, and the reticulated structure of nickel foam provided a larger surface area for electrochemical reactions, thus increasing the availability of active sites to enhance electrochemical performance. In this study, the properties were optimised through the introduction of N and P elements, and the preparation method was optimised to improve the quality and performance of the resulting materials. In comparison to the conventional synthesis method characterised by a lengthy reaction time and a significant energy expenditure, the (N, P-NiSe) rod-shaped nanocomposite electrode materials were successfully synthesised through the precise control of microwave power and microwave time. The specific capacitance of the N, P-NiSe electrode reached 3184 F g^−1^ at 1 A g^−1^ and retained 72% of its initial capacity after 4000 (10 A g^−1^) cycles. Furthermore, an asymmetric supercapacitor (ASC) was prepared with N, P-NiSe as the anode and activated carbon (AC) as the cathode. The ASC demonstrated a power density of 799.26 W kg^−1^ and an energy density of 289.06 Wh kg^−1^. Additionally, 80% of the initial capacity was retained after 20,000 cycles.

## 2. Results

### 2.1. Morphology and Microstructure

**Figure 1:**
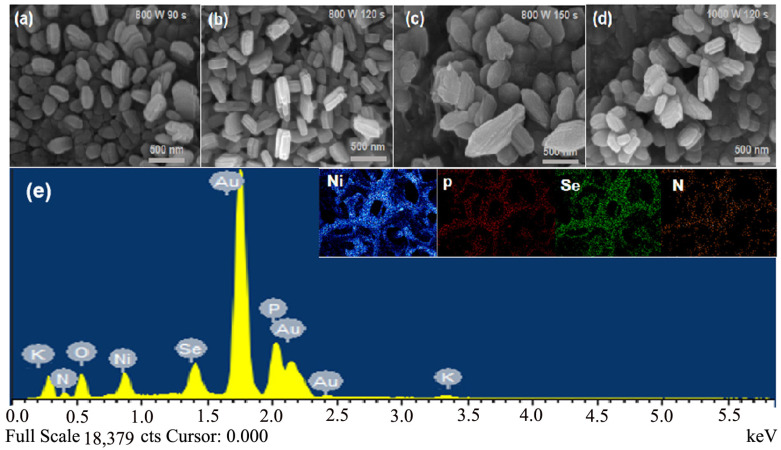
SEM images and EDS pattern of N, P-NiSe electrode material: (**a**–**d**) products prepared at 800 W 90 s (W1), 800 W 120 s (W2), 800W 150 s (W3), and 1000 W 120 s (W4), respectively; (**e**) the electronic energy pattern of the product prepared at 800 W 120 s (W2).

Figure 1a shows the SEM images of the product prepared under the following conditions: 800 W, 90 s (W1). As the microwave heating time was relatively short, the N, P-NiSe nanorods were formed less and were distributed unevenly. As illustrated in Figure 1b, with the increase in reaction time (800 W, 120 s) (W2), the nanorod particles of N, P-NiSe exhibit a uniform distribution. As shown in Figure 1c,d, when the microwave heating time is further increased or the power is increased, the rod particles aggregate on the nickel foam and eventually agglomerate into irregular shapes. The microscopic morphology of N, P-NiSe particles can be regulated by microwave power and time, with this morphology having a significant impact on electrochemical performance. The formation of agglomerates or uneven dispersion can lead to a deterioration in electrochemical performance. This is consistent with the subsequent electrochemical performance tests, where the highest capacitance values were also obtained for uniform rod-like nanomorphology observed at 800 W, 120 s (W2). The results demonstrated that the microstructure could be modulated by microwave power and time and directly affected electrochemical properties. As illustrated in Figure 1e, nitrogen (N) and phosphorus (P) were evenly distributed throughout the product, indicating successful doping. In addition, the presence of a large amount of Au elements in the EDX spectra is due to the fact that the samples were sprayed with Au in order to obtain a clearer imaging effect when performing electron microscopy. The presence of potassium is due to the use of a small amount of KOH solution to dissolve the Se powder during sample preparation, resulting in a small amount of K in the sample. Oxygen was detected because the air itself contains a large amount of oxygen.

**Figure 2:**
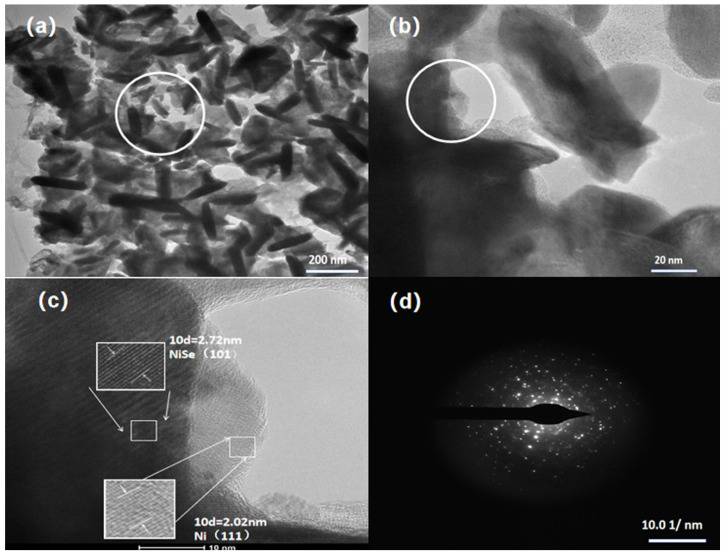
TEM images of N, P-NiSe electrode material (W2): (**a**) TEM image; (**b**,**c**) HRTEM images, (**b**) is the enlarge image labelled with a circle in (**a**), and (**c**) is the enlarge image labelled with a circle in (**b**), the bigger square in (**c**) is the enlarge image of smaller square; (**d**) a selected area electron diffraction (SAED) image.

The nanostructures within the N, P-NiSe samples (W2) were examined using TEM. As illustrated in Figure 2a, the N, P-NiSe nanorods (W2) (approximately 30 nm in width and 150–200 nm in length) are uniformly distributed on the nickel foam. The peculiar morphology and structure are further confirmed with the HRTEM images in Figure 2b,c. The lattice fringes of NiSe were 2.72 nm (101) and those of Ni were 2.02 nm (111), which is consistent with the strongest crystalline surface results of XRD. This provides further evidence of the formation of the N, P-NiSe nanorods on nickel foam. Figure 2d illustrates the result of the selected area electron diffraction (SAED) analysis, revealing the presence of dispersed bright crystalline spots, thereby confirming that N, P-NiSe is a single-crystal structure.

**Figure 3:**
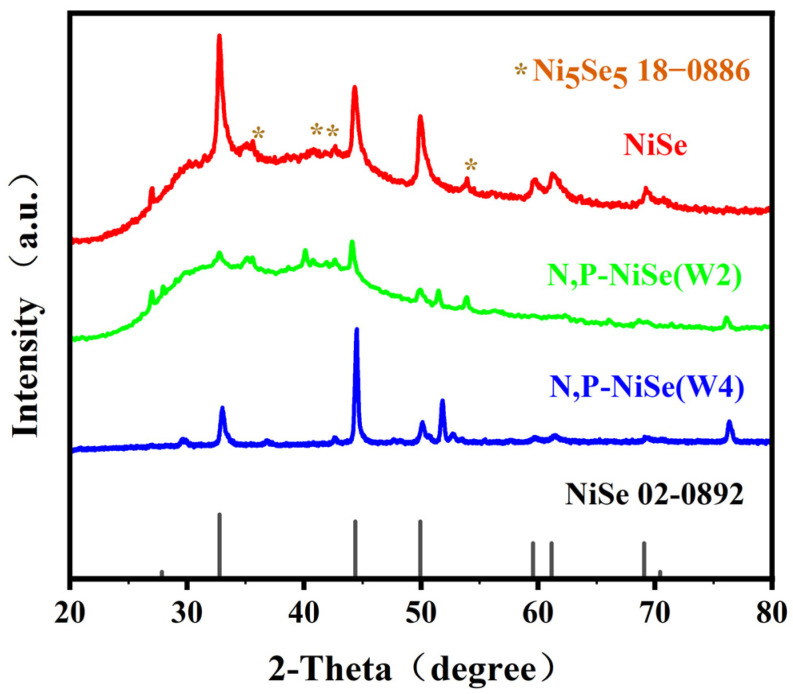
XRD patterns of N, P-NiSe electrode material (NiSe, W2, and W4).

The composition and structure of the N, P-NiSe electrode material (NiSe, W2, and W4) were investigated by X-ray powder diffraction (XRD), and the results are presented in Figure 3. The diffraction peaks of NiSe were observed at 2θ angles of 28.13°, 32.85°, 44.37°, 49.79°, 59.61°, 60.93°, and 68.87°, and could be attributed to the (100), (101), (102), (110), (103), (201), and (202) crystal faces of NiSe. The other diffraction peaks labelled with an asterisk (*) were located at 35.58°, 40.92°, 42.58°, and 53.96°, all of which can be attributed to Ni_5_Se_5_, another crystalline form of NiSe. The XRD spectra of W2 and W4 samples are also shown in Figure 3. The results show that most of the diffraction peaks can be attributed to NiSe and Ni_5_Se_5_. In addition, the impurity peaks appearing in the XRD spectra of the W2 and W4 samples (diffraction angles of 51.51° and 76.20°, respectively) were most likely owing to N and P doping.

**Figure 4:**
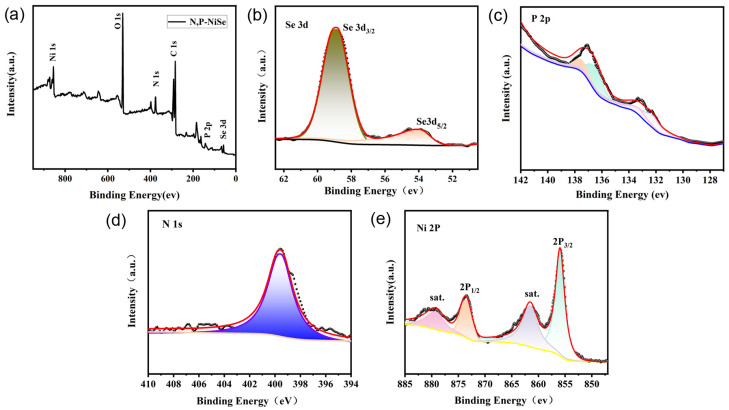
XPS patterns of N, P-NiSe electrode material (W2): (**a**) the full spectrum; (**b**–**e**) the spectra of Se, P, N, and Ni, respectively.

Further analysis of the elemental valence and elemental composition of N, P-NiSe (W2) was conducted using XPS. Figure 4a shows the full spectrum of the N, P-NiSe electrode material, which revealed the presence of Ni, Se, N, P, and C elements. Figure 4b depicts the Se 3D spectrum. The binding energies of SeOx and Se 3d_5/2_ correspond to 58.91 eV and 54.32 eV, representing the metal–selenide bond (Ni-Se Ni^2+^, Se^2−^) [27,28]. Figure 4c presents the P 2p spectrum, with binding energies at 133.4 eV and 136.8 eV. Figure 4d displays the N 1s spectrum, with the binding energy of pyrrole N corresponding to 399.5 eV, indicating the presence of both N and P elements. Figure 4e shows the spectra of Ni 2p, with binding energies at 855.83 eV and 873.35 eV for Ni 2p_3/2_ and Ni 2p_1/2_, representing the characteristics of Ni^2+^ for NiSe [27,28]. Additionally, binding energies at the satellite peaks of Ni 2p were observed at 879.16 eV and 861.74 eV, respectively. The XPS analyses indicated the successful preparation of nickel foam self-supported N, P-NiSe electrode materials.

### 2.2. Electrochemical Performance Analysis

#### 2.2.1. Electrochemical Properties of the Three-Electrode System

**Figure 5:**
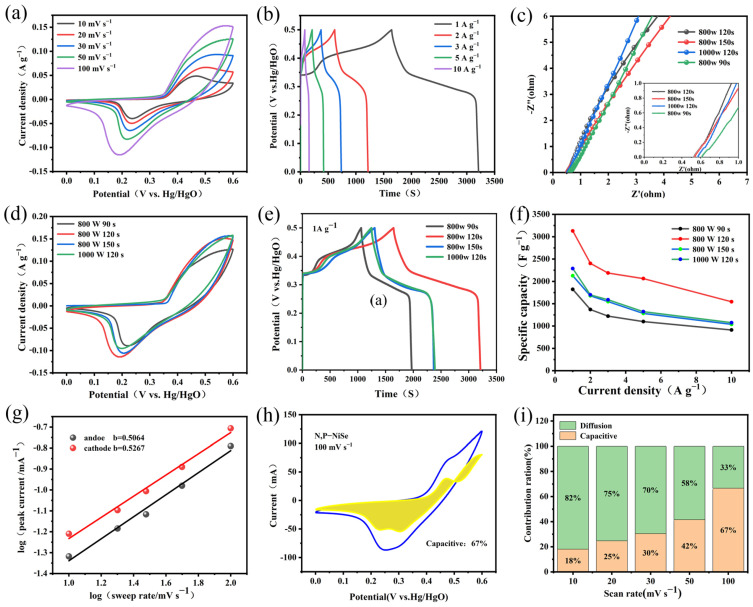
(**a**) CV curve of N, P-NiSe under optimal conditions (800 W/120 S) (W2); (**b**) GCD curve of (sample W2); (**c**) Nernquist diagram of N, P-NiSe under different reaction parameters (W1, W2, W3, and W4); (**d**) CV curves of N, P-NiSe under different microwave conditions (W1, W2, W3, and W4); (**e**) GCD curves of N, P-NiSe under different reaction parameters (W1, W2, W3, and W4); (**f**) the specific capacity of N, P-NiSe samples under different preparation conditions (W1, W2, W3, and W4); (**g**) N, P-NiSe charge storage dynamics calculation (W2); (**h**) capacity share of N, P-NiSe energy storage at 100 mV^−1^ (W2); (**i**) relative contribution of capacitance and diffusion-controlled charge storage (W2).

The electrochemical performance of the N, P-NiSe/NF electrode was tested in the three-electrode system with 6 M KOH under different conditions. The variation in the cyclic voltammetry (CV) curves for sample W2 at different scan rates is shown in Figure 5a. It can be seen in Figure 5a that the oxidation peak shifts to the right (from 0.44 to 0.56 V) and the reduction peak shifts to the left (from 0.22 to 0.17 V) as the scan rate increases. This is due to the fact that the electrode material exhibits increased resistance as the scan rate increases, which hinders the diffusion of ions and electrolytes between the electrodes. Figure 5b shows a GCD curve of sample W2. The capacity of the N, P-NiSe material was calculated to be 3184 F g^−1^ at a current density of 1 A g^−1^. A clear charge–discharge plateau can be observed in this curve, indicating that there was pseudocapacitance in the N, P-NiSe material and that it exhibited a pronounced cell-type behaviour [29]. The N, P-NiSe electrode presents a pseudocapacitive nature, and its charge storage mechanism is purely a faradaic redox reaction. Its proposed reaction mechanism is explained in [27,28,29].

NiSe+ OH^−1^ → NiSeOH + e^−1^ (1)

The differences in the internal resistance of the samples under different reaction conditions are shown in Figure 5c. Sample W2 exhibited a smaller solution resistance in the low-frequency region compared with the material under other conditions. Figure 5d shows the CV curves of N, P-NiSe materials under a variety of reaction conditions, from which it can be seen that the mathematical integral area of the CV curve is greatest for sample W2. Figure 5e illustrates the GCD curves of N, P-NiSe under different conditions. This figure also confirms that sample W2 exhibits a longer discharge time and superior Coulombic efficiency compared to the other samples. The capacitance values of samples W1, W3, and W4 were 1816 F g^−1^, 2130 F g^−1^, and 2242 F g^−1^, respectively, which were in agreement with the results of CV tests. Figure 5f presents the line graphs of the capacitance values of N, P-NiSe at different current densities for different conditions. It can be observed that 120 s has the highest capacitance value regardless of the condition of 800 W. The charge storage mechanism of N, P-NiSe material was investigated by fitting and calculating cyclic voltammetry curves [30]. The relationship between the peak current (I) and the scan rate (v) was determined using the following formula:

I = a × v^b^ (2)

where b is the slope of the fitted straight line. If b = 1, it can be concluded that a non-Faraday process controls charge storage. Conversely, if b = 0.5, it can be inferred that a Faraday process controls charge storage. As shown in Figure 5g, the fitted b values for the anodic oxidation and cathodic reduction peaks of N, P-NiSe were 0.50554 and 0.5267, respectively. This finding suggests that diffusion-controlled Faraday processes (cell behaviour) dominate charge storage. Furthermore, the total charge storage contribution can be determined using the following equation:

i (v) = k_1_v + k_2_v^1/2^ (3)

where k_1_v represents the surface control capacitance, and k_2_v^1/2^ is the diffusion control capacitance [31]. As shown in Figure 5h, the surface control capacitance accounted for 67% of the total capacity at 100 mV s^−1^. Figure 5i illustrates the capacitance occupancy at varying sweep rates, demonstrating that the capacitive behaviour dominates the charge storage mechanism of N, P-NiSe/NF as the sweep rate increases. The doping of the N, P elements provides a plethora of active sites for electron storage, endowing the N, P-NiSe/NF with excellent reversible pseudocapacitive charge storage capability. 

In addition, with the preparation condition of 800 W and 120 S, we performed comparison experiments with the dosage of 5 mg, 10 mg, and 15 mg of hexachlorocyclotriphosphonitrile as the dopant precursor and found that the total amount of N and P doping could be adjusted, but due to the small amount, it was difficult to determine the accurate total amount of N and P doping directly. In order to explore the effect of N and p doping on the electrochemical performance of the electrodes, cyclic voltammetry and galvanostatic charge–discharge tests were carried out on the samples with different dopant precursor amounts (as shown in Figure S1, Supplementary Materials). The specific capacitance values for precursor doping amounts of 5 mg, 10 mg, and 15 mg were 2340, 3184, and 2280 F g^−1^, respectively, indicating that the optimal precursor doping amount was 10 mg.

**Figure 6:**
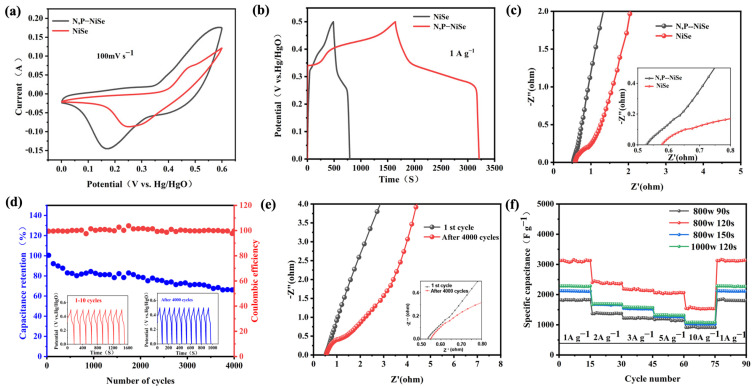
(**a**) CV curves of N, P-NiSe (W2) and NiSe; (**b**) GCD curves of N, P-NiSe (W2) and NiSe; (**c**) EIS plots of N, P-NiSe (W2) and NiSe; (**d**) capacitance retention and Coulomb efficiency; (**e**) EIS plots before and after cycling (W2); (**f**) multiplicity performance of sample (W1, W2, W3, and W4).

The electrochemical properties of N, P-NiSe electrode materials and the undoped single NiSe were compared. The cyclic voltammetry (CV), galvanostatic charge–discharge (GCD), and electrochemical impedance spectroscopy (EIS) plots of the NiSe and N, P-NiSe (W2) materials are shown in Figure 6a, Figure 6b, and Figure 6c, respectively. There is a significant difference between the two CV plots at 100 mV s^−1^. The N, P-NiSe integral area is larger than that of NiSe, indicating that N, P doping improves the electrochemical performance. In comparison to the maximum specific capacitance value of NiSe (514 F g^−1^ at 1 A g^−1^), the capacitance value of N, P-doped NiSe reached 3184 F g^−1^ at the same current density. This can be attributed to the distortion of the lattice of the electrode material caused by the doping of N, P elements, which generates more electrochemically active sites and thus increases the capacitance value. The introduction of electrochemically active sites, which increased the capacitance value dramatically, is evident in the electrochemical impedance spectroscopy (EIS) impedance plots. The resistance (Rs) of the N, P-doped NiSe electrode material and that of the NiSe electrode material were 0.53 Ω and 0.58 Ω, respectively. This indicates an increase in conductivity after doping, which is consistent with the large capacitance values obtained for N, P-doped NiSe. As illustrated in Figure 6d, the N, P-NiSe electrode material exhibits excellent cycling stability, with a capacitance retention of above 72% following a 4000-cycle performance test at 15 A g^−1^. Figure 6e illustrates that, despite the notable enhancement in stability, it is still possible that the internal volume of N, P-NiSe underwent expansion due to repeated charging and discharging cycles. This may have resulted in the irreversible oxidation of some electrodes, which led to a decline in performance. Furthermore, a slight increase in the internal resistance was observed. The multiplicative performance of N, P-NiSe is demonstrated in Figure 6f, which illustrates that after multiple charging and discharging cycles at different current densities, the capacitance retention of N, P-NiSe remains at more than 72% after 4000 cycles. The capacitance value of sample W2 was maintained at 3184 F g^−1^ at 1 A g^−1^ after several charge–discharge cycles, indicating that the material has good multiplicative properties. A comparison of N, P-NiSe with the same type of material is presented in Table 1.

#### 2.2.2. Electrochemical Properties of the Two-Electrode System

**Figure 7:**
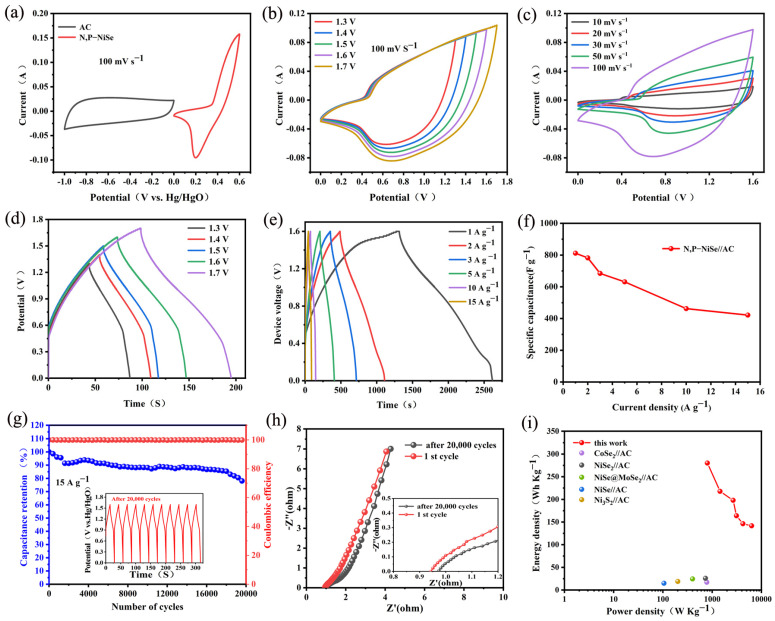
(**a**) CV curves of N, P-NiSe (W2) and AC; (**b**) CV curves at different voltages; (**c**) CV curves of N, P-NiSe//AC at different scanning rates at 1.6 V; (**d**) variable current GCD curves of N, P-NiSe//AC at different voltages; (**e**) GCD curves of N, P-NiSe//AC AC at different current currents; (**f**) line chart of capacitance value of N, P-NiSe//AC at different current densities; (**g**) cycle stability and coulomb efficiency after 20,000 cycles; (**h**) EIS image before and after 20,000 cycles; (**i**) energy–power density diagram of different devices.

In order to explore the performance of the N, P-NiSe electrode material in greater depth, sample W2 and activated carbon were combined to form an asymmetric supercapacitor. Figure 7a–d illustrate the electrochemical performance of the N, P-NiSe//AC supercapacitor, which comprises sample W2 and activated carbon in a liquid electrolyte. The mass ratios of the AC electrode and the N, P-NiSe (sample W2) electrode were adjusted according to Equation (4). Figure 7a illustrates the cyclic voltammetry (CV) curves of the AC electrode (operating at −1 to 0 V) and the N, P-NiSe electrode (operating at 0 to 0.6 V) in the N, P-NiSe//AC configuration. This configuration was based on the complementary relationship between the working electrodes, which comprise a bilayer and exhibit pseudocapacitance. Figure 7b shows the CV curves of N, P-NiSe//AC, with no discernible distortion, indicating that it has good current responsiveness. N, P-NiSe//AC was capable of functioning properly at 1.6 V. Figure 7c illustrates the CV curves of N, P-NiSe//AC at 10–100 mV s^−1^. Figure 7d shows the constant current GCD curves at different voltages (1.3–1.7 V). It can be observed that the shape of the curve changes at 1.7 V, which may be attributed to electrode polarisation. Consequently, the optimum operating voltage window for this device was 1.6 V. Figure 7e illustrates the constant current GCD curves at varying current densities. Figure 7f presents a line graph of the capacitance values at different current densities. Figure 7g depicts the cycling performance of N, P-NiSe//AC, demonstrating that the capacitance retention remains high even after 20,000 consecutive charge–discharge cycles. Figure 7h illustrates the change in the internal resistance of the solution following 20,000 cycles. The *p*-value of N, P-NiSe//AC was 289.06 W kg^−1^, and the corresponding E-value was 799.26 Wh kg^−1^, indicating that the N, P-NiSe//AC has a high energy density. The Ragone diagrams of N, P-NiSe//AC with other electrode materials are shown in Figure 7i. Furthermore, the E-value of N, P-NiSe//AC is demonstrably higher than that of other related materials reported in the literature [32,33,34,36,37], thereby indicating that the electrochemical performance of N, P-NiSe//AC is superior.

## 3. Materials and Methods

### 3.1. Material Preparation

Nickel foam (NF) was purchased from Long Sheng Bao Products Company (Guangzhou, China). Prior to the experiment, NF was ultrasonically cleaned with hydrochloric acid, ethanol, and ultrapure water to ensure that it was free of impurities. It was then vacuum-dried at 80 °C. Selenium powder was purchased from Aladdin Industries. The hexachlorocyclotriphosphonitrile (HCCP) was sourced from Leyan (Shanghai, China). Anhydrous ethanol, ethylene glycol (EG), ethylenediamine (EDA), and potassium hydroxide (KOH) were procured from the Tianjin Damao Chemical Company (Tianjin, China).

### 3.2. Preparation of N, P-NiSe/Ni

**Figure 8:**
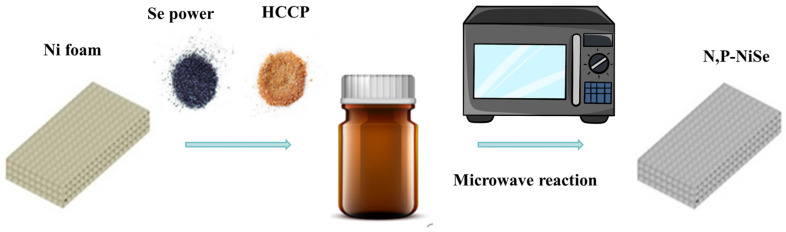
The preparation process of N, P-NiSe/Ni.

Briefly, 20 mg of selenium powder, 10 mg of hexachlorocyclotriphosphonitrile powder, 300 µL of potassium hydroxide (6 M), 2 mL of C_2_H_8_N_2_, and 1 mL of C_2_H_6_O were dissolved by stirring to obtain a purplish-black liquid. Subsequently, nickel foam was immersed in the solution and sonicated for 15 min. The foam was then transferred to a heat-resistant crucible (10 mL). After being kept in a microwave oven at a certain power and time, the nickel foam was washed with deionised water and anhydrous ethanol and further dried (80 °C, 12 h). The preparation process is illustrated in Figure 8, and the different reaction conditions are presented in Table 2.

### 3.3. Preparation of N, P-NiSe/Ni//AC

N, P-NiSe/Ni was employed as the positive electrode, while a 1 × 1 cm^2^ commercial AC electrode served as the negative electrode, and the resulting device was tested in 6 M potassium hydroxide. The negative electrode was prepared as follows: Activated carbon, acetylene black, and PVDF were mixed into a homogeneous slurry in a mass ratio of 80:10:10. The slurry was uniformly applied to 1 cm^2^ NF, dried at 100 °C for 10 h, and finally pressed into the electrode sheet.

### 3.4. Characterisation of N, P-NiSe

The crystal structure of the resulting samples was characterised by powder X-ray diffraction (XRD). The surface morphology and microscopic internal structure of the samples were determined by scanning electron microscopy (SEM) and transmission electron microscopy (TEM). X-ray photoelectron spectroscopy (XPS) and energy-dispersive spectroscopy (EDS) were used to identify the elemental composition and distribution of the samples.

### 3.5. Electrochemical Testing

The electrochemical properties of N, P-NiSe composites were tested by cyclic voltammetry (CV), constant current charge–discharge (GCD), and electrochemical impedance spectroscopy (EIS) in 6 M potassium hydroxide. A platinum sheet was used as the counter electrode, and a mercuric oxide electrode was used as the reference electrode. An electrochemical workstation (CHI660E) was utilised for electrochemical data testing. The cycling stability of the materials was evaluated using the Landt battery test system (CT2001A). The capacitance values were calculated using the following equation:

C (F g^−1^) = I Δt/(m ∆V) (4)

A two-electrode test system was assembled with AC as the negative electrode and the synthetic material as the positive electrode. In this case, the mass ratio between the positive and negative electrodes was determined with the following equation:

m^+^⁄m^−^ = C^−^ ΔV^−^/C^+^ΔV^+^ (5)

The energy density (E) and power density (P) were calculated using the following equations:

E = C ΔV^2^/7.2 (6)

P = 3600 E/Δt(7)

## 4. Conclusions

In summary, N, P-NiSe composites were prepared using the microwave method, exhibiting excellent capacitance performance and remarkable capacitance retention at high current density and long operation time. The synergistic interaction between N, P and NiSe resulted in the excellent electrochemical properties of N, P-NiSe. The capacitance values of the material were up to 3184 F g^−1^ (1 A g^−1^), and it was observed that 72% of the original capacitance value was maintained through 4,000 charge–discharge cycles. Furthermore, the practical application of the electrode was demonstrated through the assembly of devices comprising N, P-NiSe//AC, which exhibited an energy density of 289.06 W h kg^−1^ and a power density of 799.26 W kg^−1^ and retained 80% of the initial capacitance after 20,000 cycles. These outcomes not only offer novel insights into the optimisation of NiSe but also facilitate the advancement of novel supercapacitors.

## Figures and Tables

**Table 1:** Comparison of electrochemical properties of N, P-NiSe (W2) with other materials.

Material	Method	Specific Capacity	Retention Rate	Ref.
NiSe microspheres	Hydrothermal	492 F g^−1^ (0.5 A g^−1^)	200 cycles, 84.6%	[32]
NiSe_2_@rGO	Microwave	580 F g^−1^ (1 A g^−1^)	5000 cycles, 78.13%	[33]
NiSe@MoSe_2_	Hydrothermal	223 Fg^−1^ (1 g^−11^)	1000 cycles, 93.7%	[34]
Ni_0.85_Se@MoSe_2_	Hydrothermal	774 g^−1^ (1 A g^−1^)	5000 cycles, 88%	[35]
NiSe@RGO	Hydrothermal	781 C g^−1^ (1 A g^−1^)	5000 cycles, 90%	[26]
N, P-NiSe	Microwave	3184 F g^−1^ (1 Ag^−1^)	4000 cycles, 72%	This Work

**Table 2:** Specific capacitance values of N, P-NiSe/Ni under different reaction conditions.

Sample	Microwave Watts/W	Microwave Duration/s	Duration/s Capacitance/F g^−1^ (1 A g ^−1^)
W1	800 W	90 S	1816 F g^−1^
W2	800 W	120 S	3184 F g^−1^
W3	800 W	150 S	2130 F g^−1^
W4	1000 W	120 S	2242 F g^−1^

## Data Availability

The data presented in this study are available on request from the corresponding author.

## Supplementary Materials

The following supporting information can be downloaded at: https://www.mdpi.com/article/10.3390/molecules29133224/s1. Figure S1 (a) cyclic voltammetry curve and (b) galvanostatic charge-discharge curve of different N, P dopant precursor contents.
