# Supplementary material for: The Preparation of N, P-Doped NiSe Nanorod Electrode Materials on Nickel Foam Using the Microwave Method for High-Performance Supercapacitors
Source: Molecules. 2024 Jul 7;29(13):3224. doi: 10.3390/molecules29133224 (PMC11243357; doi:10.3390/molecules29133224)
Supplement: Supplementary file 1 [file molecules-29-03224-s001.zip › molecules-3074952-supplementary.pdf]

The Preparation of N, P-Doped NiSe Nanorod Electrode Materials on Nickel Foam Using the Microwave Method for High-Performance Supercapacitors

Zhen Lu[1], Hongjie Kang[1], Qianwen Duan[1], Chao Lv[2], Rui Liu[1], Feng Feng[1,*], Haidong Zhao[1,*]

[1] School of Chemistry and Chemical Engineering, Shanxi Datong University, Datong, 037009, China
[2] School of Coal Engineering, Shanxi Datong University, Datong, 037009, China
* Correspondence: feng-feng64@263.net (F. F.); zhaohd@sxdtdx.edu.cn (H. Z.)

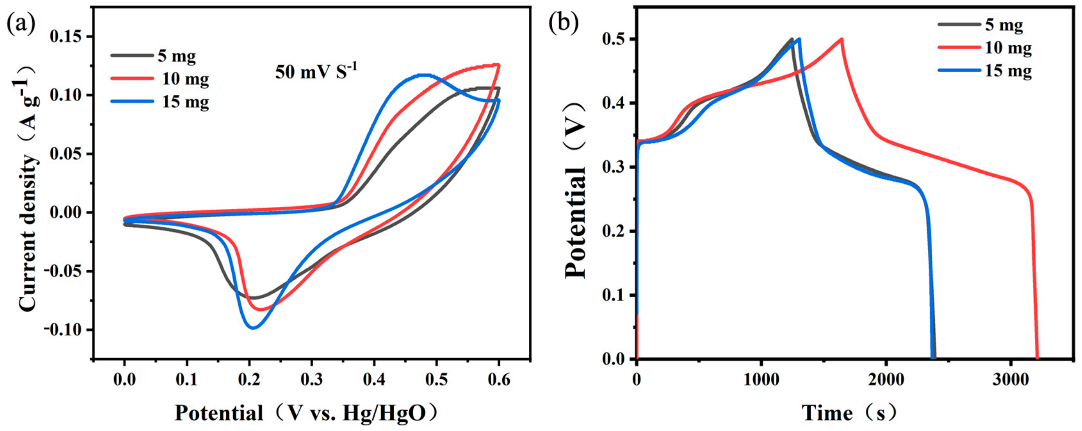

Fig. S1 (a) cyclic voltammetry curve and (b) galvanostatic charge-discharge curve of different N,P dopant precursor contents.
